# Gata3 is Required in Late Proneurosensory Development for Proper Sensory Cell Formation and Organization

**DOI:** 10.21203/rs.3.rs-2747944/v1

**Published:** 2023-04-14

**Authors:** Paige V. Blinkiewicz, Makayla R. Long, Zachary A. Stoner, Elizabeth M. Ketchum, Sydney N. Sheltz-Kempf, Jeremy S. Duncan

**Affiliations:** Western Michigan University; Western Michigan University; Western Michigan University; Western Michigan University; Western Michigan University; Western Michigan University

## Abstract

It has been previously shown that zinc-finger transcription factor *Gata3* has dynamic expression within the inner ear throughout embryonic development and is essential for cochlear neurosensory development. However, the temporal window to which *Gata3* is required for the formation of the cochlear neurosensory epithelia remains unclear. To investigate the role of *Gata3* on cochlear neurosensory development in the late prosensory stages, we used the *Sox2-cre*^*ERT2*^ mouse line to target and conditionally delete *Gata3* at E11.5 before the cells have fully committed to a neurosensory fate. While the inner ears of *Sox2-cre*^*ERT2*^*: Gata3 f/f* mice appear morphologically normal, the sensory cells in the organ of Corti are partially lost and disorganized in a basal to apical gradient with the apex demonstrating the more severe phenotype. Additionally, spiral ganglion neurons display aberrant peripheral projections, such as increased distances between radial bundles and disorganization upon reaching the organ of Corti. Furthermore, heterozygous *Sox2-cre*^*ERT2*^*: Gata3 f/+* mice show a reduced phenotype in comparison to the homozygous mutant, supporting the concept that *Gata3* is not only required for proper formation at the later proneurosensory stage, but also that a specific level of *Gata3* is required. Therefore, our studies confirm that *Gata3* plays a time-sensitive and dose-dependent role in the development of sensory cells in the late proneurosensory stages.

## Introduction

The mammalian inner ear is comprised of six unique sensory organs, but only one of these organs is responsible for the sense of hearing: the cochlea. The cochlea contains the organ of Corti (OC) which is comprised of mechanosensory hair cells (HCs) and their corresponding supporting cells (SCs). HCs transduce sound energy into electrical impulses via innervation by spiral ganglion neurons (SGNs) that project into the hindbrain for further auditory processing. The development of these three sensory cell types has been extensively studied, but there are still gaps in knowledge regarding the transcription factors and gene networks that control the spatial and temporal aspects of this process at later proneurosensory stages.

The inner ear is derived from the otic placode, which will invaginate to form the otic cup before developing into the otocyst around embryonic day 9 (E9)^1,2^. While several transcription factors are important for neurosensory development in this time frame, the zinc-finger transcription factor *Gata3* is particularly interesting due to its dynamic expression throughout development. While *Gata3* is initially expressed as early as E8.5 throughout the otocyst, by E10.5 its expression is restricted to the proneurosensory regions^3–9^. *Gata3* continues to be expressed in SGNs until postnatal day 14 (P14) and remains highly expressed in SCs, with lower levels in HCs, throughout adulthood^10–14^.Therefore, it has been postulated that *Gata3* plays an important and dynamic role in inner ear development and neurosensory cell formation during this embryonic temporal window.

Previous studies have shown that loss of *Gata3* in the early proneurosensory region around E8.5 leads to loss of all cochlear neurosensory cells^5,15^, while loss of *Gata3* one day later around E9.5 leads to a patchy loss of HCs and SCs, and disorganization and patchy loss of SGNs. Other studies have investigated the role of *Gata3* postnatally in the maintenance of HCs and SCs^11,12^. These studies found that *Gata3* is necessary later on to maintain OHCs and to functionally develop IHCs, while loss of *Gata3* from postnatal SCs results in an increase in some types of SCs through downregulation of other genes. However, there exists a gap in knowledge about the role of *Gata3* later in embryonic development as the proneurosensory cells start to differentiate into HCs, SCs, and SGNs. Specifically, it remains to be seen how long expression is required for proper embryonic development of neurosensory cells before *Gata3* switches to its postnatal maintenance role. Additionally, while we know the presence of *Gata3* is necessary for proper neurosensory development, the precise level of *Gata3* expression is also important for maintenance and function. For example, both *Gata3* haploinsufficiency and *Gata3* over-expression, as a result of gene duplication, cause human hypoparathyroidism, sensorineural deafness, and renal dysplasia (HDR) syndrome^16–21^. While the triad of symptoms of HDR syndrome range in severity, nearly all patients exhibit deafness^16,19,22,23^. Uniquely, deafness is the only symptom of HDR syndrome which can present singularly^16,19,21,23^. This suggests that not only is continued expression of *Gata3* required for proper inner ear development, but specific levels of *Gata3* are also required. Continued investigation of the dose-dependent requirements of *Gata3* would also contribute to the fields’ overall understanding of inner ear gene regulatory networks.

Therefore, in this study, we explored the window of developmental plasticity which is governed by *Gata3* as a follow up to previous studies showing its loss is detrimental to cochlear neurosensory epithelia^5–7, 10,21,24,25^. Using the *Sox2-cre*^*ERT2*^ mouse line^26^, we conditionally deleted *Gata3* from proneurosensory cells via tamoxifen (TMX) injection at E11.5. Our results show that deletion of *Gata3* causes severe loss and disorganization of HCs, SCs, and SGNs in a basal to apical gradient, with a more severe phenotype presenting in the apex. Interestingly, the mutant ears were morphologically normal in that it presented with a full-length cochlea unlike previous *Gata3* deletion studies. Overall we show that, while *Gata3* is not necessary at E11.5 for overall morphological development and elongation of the cochlea, however, *Gata3* is required in the late proneurosensory stages for cochlear sensory epithelia and SGNs both to form and organize properly.

## Results

### Gata3 is deleted from HCs, SCs, and SGNs at E11.5

**Figure 1:**
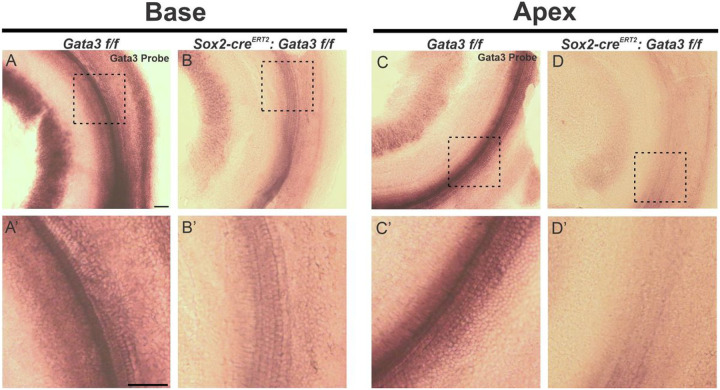
Gata3 is conditionally deleted from HCs, SCs, and SGNs at E11.5 (**A-D’**) Whole mount *in-situ* hybridization was performed with a *Gata3* probe on a *Gata3 f/f* control and a *Sox2-cre*^*ERT2*^*: Gata3 f/f* mutant and imaged at the cochlear base and apex. *Gata3* expression appears in the HCs, SCs, and SGNs of the control and is absent in the HCs and SCs and decreased in the SGNs of the homozygote mutant. Scale bar: 100 μm

Previous studies have characterized *Sox2-cre*^*ERT2*^ expression at the placode stage (E8.5), otocyst stage (E10.5), and the late otocyst stage (E12.5)^27–32^. At E10.5, *Sox2* is present in both the nonsensory cochlear floor and roof^31^. By E12.5, *Sox2* is exclusively expressed in OC sensory cells^31^. In order to confirm complete knockout of *Gata3* from HCs, SCs, and SGNs, *in situ* hybridization was performed using a *Gata3* riboprobe. While the control shows high expression of *Gata3* in all cell types from base to apex, the homozygous mutant shows no expression in the HCs and SCs and greatly reduced expression in the SGN cell bodies (Fig. 1A–D’), demonstrating that our model is indeed reducing levels of *Gata3* in the cell types of interest.

### Gata3 is required for sustained formation and organization of HCs

**Figure 2:**
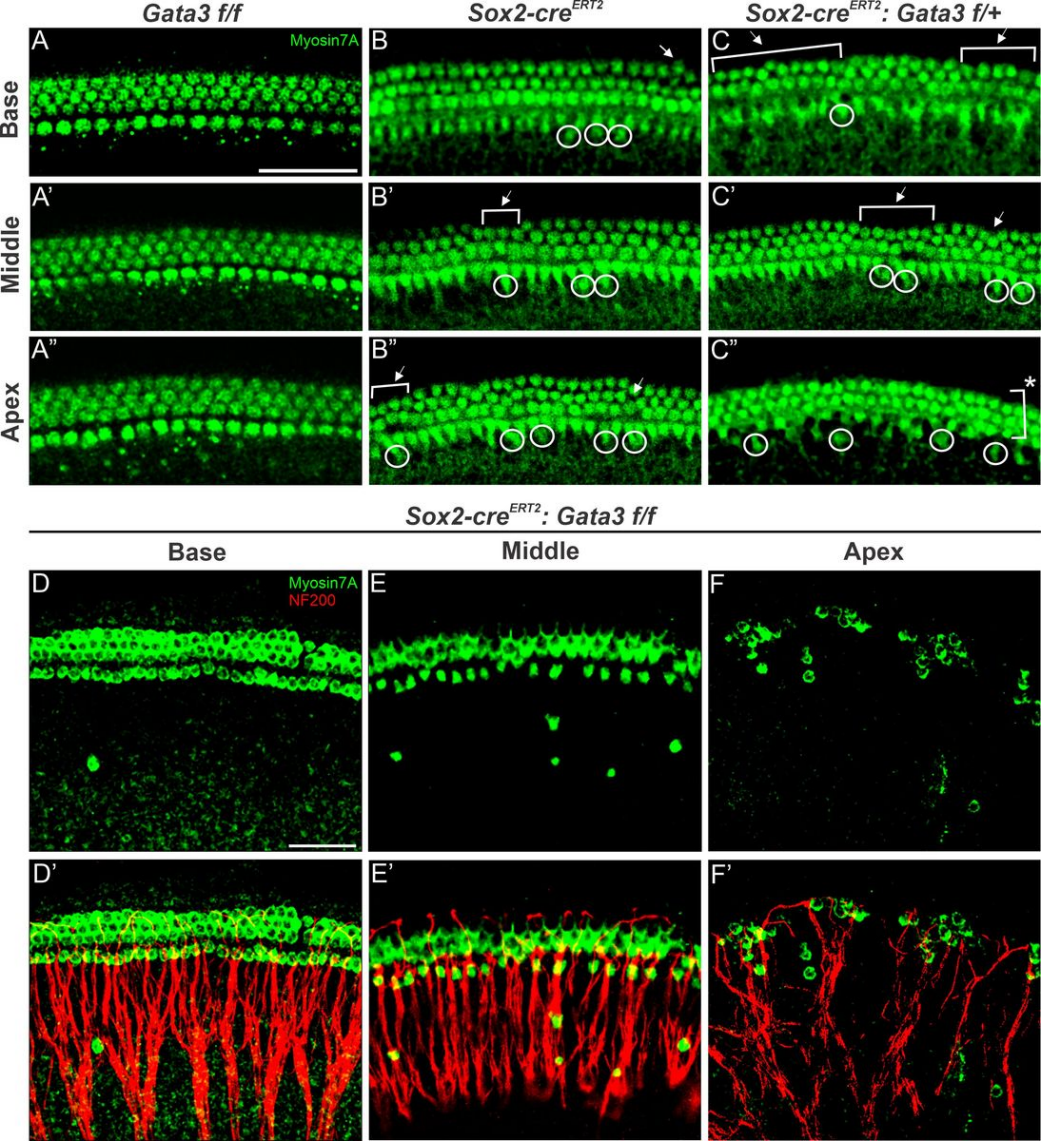
Deletion of *Gata3* results in loss of HCs in a basal to apical gradient (**A-F’**) Representative images from the basal, middle, and apical regions of the cochlea for HCs indicated by MYOSIN7A+ staining. Two different controls were used, *Gata3 f/f* and *Sox2-cre*^*ERT2*^, in order to account for the haploinsufficent phenotype of the Cre line used. Both the heterozygous and homozygous mutant show IHC duplets (white circles) and missing rows of OHCs (white brackets), while the homozygous mutant also shows ectopic HCs in the GER. Scale bar: 50 μm

We first analyzed the effect of deletion of *Gata3* on HCs, as previous *Gata3* CKOs show either no HC development or only patches of HCs^5–7, 10^. In order to assess the phenotype of the deletion of *Gata3*, two different controls were used: *Gata3 f/f* (Fig. 2A–A”) and *Sox2-cre*^*ERT2*^ (Fig. 2B–B”). Other studies have previously demonstrated that the knock-in *Sox2-cre*^*ERT2*^ line shows inner hair cell (IHC) duplets, which was confirmed in our study (Fig. 2B–B”; white circles). It was important to investigate the IHC duplets in the heterozygous mutant compared to the *Sox2-cre*^*ERT2*^ control to ensure that the phenotype seen is not the result of using this Cre line (Fig. 2C–C”). While the base, middle and apex of the heterozygous mutant all contain IHC duplets similar to the *Sox2-cre*^*ERT2*^ control, it should be noted that the third row of outer hair cells (OHCs) is lost in the middle region of the OC into the apical region (Fig. 2C–C”). We found that the heterozygous genotype shows a continuous formation of HCs from base to apex, while the homozygous mutant shows some disturbances in the apical region, similar to the previous *Gata3* CKO study that showed the presence of patches^6^. The homozygous mutant also shows a worsening phenotype compared to the heterozygous mutant. While the base contains all three rows of OHCs, progressive rows of OHCs are lost (Fig. 2D–D’). Just two rows of OHCs are present in the middle region and almost no rows of OHCs are present in the apex (Fig. 2E–F”). Additionally, the entire cochlea contains Myosin VIIa positive cells in the GER with the highest number appearing in the apex, which is similar to a postnatal *Gata3* CKO from SCs using this same Cre line^11^. Furthermore, these cells in the GER are associated with SGN endings. Ectopic HCs have been seen in the GER in both CKO and over-expressor models previously^33–38^. While ectopic HCs generally are not seen in combination with missing rows of OHCs, previous studies have shown that loss of *Gata3* results in missing OHCs postnatally^12,21^. The phenotype of both ectopic HCs and missing rows of OHCs as a result of embryonic loss of *Gata3* is unique and further supports a role for *Gata3* in this specific temporal window in this specific cell type.

### Gata3 is required for corresponding SC formation and organization

**Figure 3:**
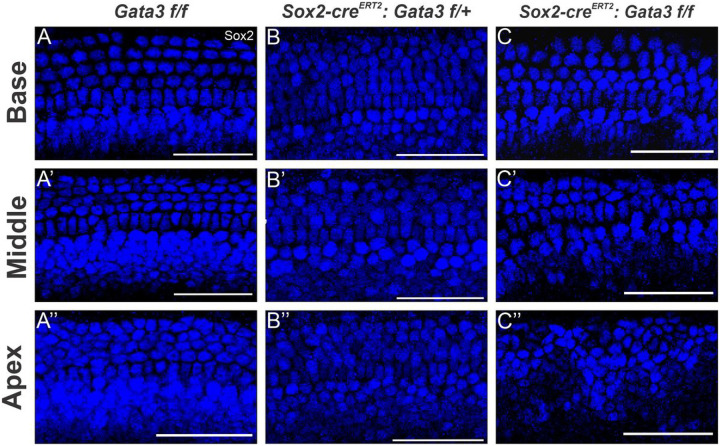
Deletion of *Gata3* results in loss of SCs in a basal to apical gradient (**A-C’**) Representative images from the basal, middle, and apical regions of the cochlea showing SCs indicated by SOX2+ staining. The homozygous mutant shows a worsening disorganization of SCs from base to apex, with entire rows of SCs missing in the middle and apex. Scale bar: 25 μm

Previous studies that have deleted *Gata3* have shown either no SC development or only limited patches of SC formation localized to the HC patches^5,6,10^. However, we still observe SCs in our model throughout the majority of the length of the cochlea. Similar to the HC phenotype in this model, we found that the homozygous mutant shows almost continuous formation of SCs, except for some patches in the apex (Fig. 3). The apex also shows disorganization of the SC rows. The homozygous mutant shows a worsening phenotype compared to the heterozygous mutant, similar to that seen in the HCs. The base and middle show disorganized SCs and complete loss of some outer SC rows in the middle region. The apex contains the most severe phenotype in which SC appear to cluster together which is very similar to the SC phenotype seen in other *Gata3* CKO studies^10^. Ultimately, the phenotype in the HCs and SCs are consistent in their appearance of progressive loss of OHCs from base to apex, mirroring the loss of SCs from base to apex in the homozygous mutant. While this SC disorganization in our model is also similar to the phenotype seen in other *Gata3* CKO studies, it is important to note that the previous study did not also observe ectopic HCs in the GER^10^. Further studies are needed in order to tease apart the specific requirement for *Gata3* within this specific time window to determine if *Gata3* deletion in one cell population can influence another cell population.

### Gata3 Is Required For Organization Of Sgn Peripheral Projections

**Figure 4:**
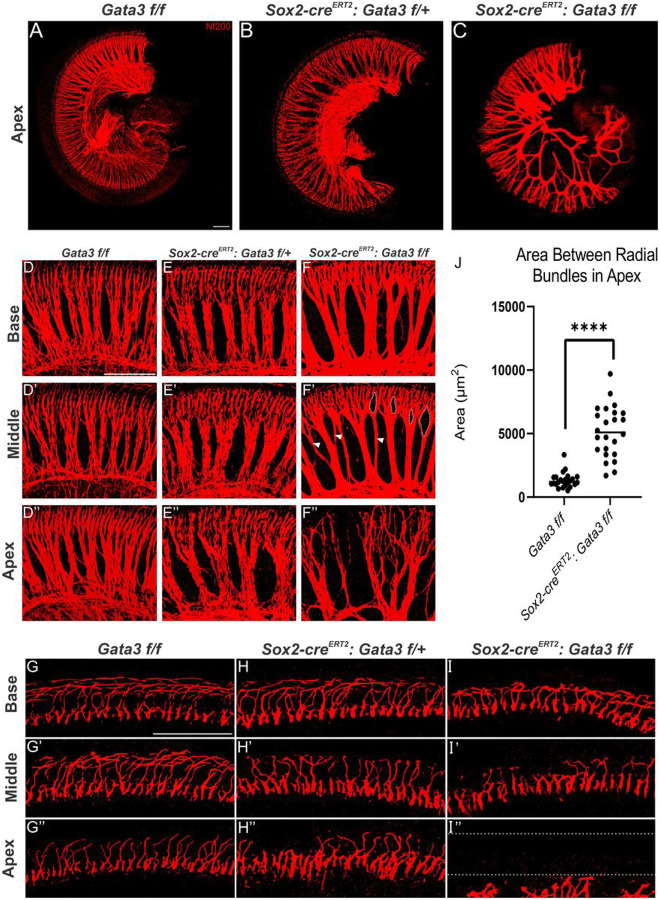
Deletion of *Gata3* results in fewer of SGNs in a worsening gradient from base to apex (**A-C**) Overview of SGNs in the control, heterozygous mutant, and homozygous mutant apex. (**D, D’, D”**)Radial bundles of a control sample. (**E, E’, E”**) Radial bundles of a heterozygous mutant. Slightly increased space between the radial bundles is observed in the apex. (**F-F”**) Radial bundles of a homozygous mutant. The distance between radial bundles is increased relative to the control sample. The dotted white outline and white arrowheads in **F’** indicate increased branching in the middle. The radial bundles in **F”** exhibit even greater amounts of branching as well as an increase and irregular distance between the fibers. (**F”**) (**G, G’, G”**) Peripheral projections of the control as the reach the OC are well organized in the control. (H) In the heterozygous mutant, all peripheral neurites are present and relatively organized. (**H’**) Peripheral neurites in the middle of the heterozygous mutant have some peripheral projections misturn towards the apex instead of the base. Additionally, there are fewer neurites present than in the control. **(H”**) In the apex of the heterozygous mutant, there are fewer peripheral projections and those that are present show disorganization relative to the control sample. (**I)** In the base of the homozygous mutant, peripheral projections are present but are fewer in number and show an increased number of neurites turning towards the apex rather than the base. (**I’**)The middle of the homozygous mutant has drastically fewer neurites reaching the OC relative to the control, particularly those neurites that should project to the OHC region. (**I”**) The apex of the homozygous mutant has some peripheral neurites approaching the IHC region of the OC but no peripheral neurites extending to the OHC region. (**J**)Quantification of the distance between radial bundles in apex of control samples versus apex in homozygous mutants. The distance between radial bundles is greater in mutant samples than in controls and mutant samples show greater variability in the distance between radial bundles. A TTEST was performed and p < 0.0001. Scale bar: 100 μm

Our observation of Myosin VIIa positive cells associated with SGN endings in the homozygous mutant led us to investigate the peripheral projections of SGNs to confirm that they were developing properly. Previous studies examining the effect of *Gata3* deletion from the proneurosensory region of the developing otocyst observed a severe reduction in the number of SGNs present in mutant samples, while the SGNs that did form displayed aberrant projection patterns towards the developing OC^6,10^. Another study in which *Gata3* deletion was restricted to SGNs saw proper formation of SGNs with disorganized peripheral projections^14,24^. We first examined peripheral projections in a *Sox2-cre*^*ERT2*^ mutant sample to establish whether the Cre knock-in displays a SGN phenotype. When compared with control samples (Fig. 4D–D”), we saw no obvious difference in SGN number or organization (data not shown). We then looked at the peripheral projections in a heterozygous mutant. The number and overall organization of SGNs in the heterozygous mutant largely resemble control samples in the base and middle (Fig. 4E–E’). However, the radial bundles in the apex of the heterozygous mutant appear to have an increased area separating them relative to the control (F”). The homozygous mutant has a striking phenotype that displays an increase in the distance between radial bundles as well aberrant projections of the radial bundles which becomes progressively more disorganized along the length of the cochlea (Fig. 4F–F”). The mutant base and middle (Fig. 4F–F’) reveal irregular distances between radial bundles in addition to extra branches from radial bundles. There are also inconsistencies in their organization, as some gaps are large and others are reduced (Fig. 4C). This phenotype is even more profound in the apex (Fig. 4F”).

The area between the radial bundles in the apex of control and homozygous mutant samples was measured and quantified (Fig. 4J). The method for radial bundle distance quantification can be found as Supplementary Fig. S1 online. Analysis of the data showed a statistically significant increase in the distance between radial bundles in the homozygous mutant relative to the control (p < 0.0001). Additionally, the values for the area in mutant samples was highly variable, further supporting that loss of *Gata3* results in disorganization of peripheral projections of SGNs.

We also examined the peripheral projections where the neurites reach the OC (Fig. 4G–I”). The basal region of the heterozygous mutant is comparable to the control (Fig. 4G–H), but peripheral projections are progressively fewer and become disorganized in the heterozygous mutant with progression to the middle and apex (Fig. 4G’–G”, H’–H”). Peripheral projections in the base of the homozygous mutant appear slightly disorganized upon reaching the OC. Additionally, the density of neurites in the homozygous mutant appears to be less when compared to the base of the control (Fig. 4I). The disorganization of the neurites and decreased density is even more pronounced in the middle and apex of the homozygous mutant (Fig. 4I’–I”). Fewer neurites project into the OHC region of the OC in the middle and few-to-no neurites project to the OHC region in the apex. In these regions, not all neurites that are present within the OHC region properly turn towards the base but rather, turn towards the apex.

Based upon our results, *Gata3* expression is important for the formation of radial bundles with regards to appropriate density and distance between bundles, as well as for proper branching patterns and overall organization. Additionally, *Gata3* is needed for peripheral neurites to reach the OC, particularly the OHC region, and to form proper connections with HCs. Importantly, the loss of *Gata3* has a phenotype that progressively worsens along the length of the cochlea, with the greatest phenotype observed in the apex.

### Gata3 Is Required For Proper Central Pathfinding Of Sgns

**Figure 5:**
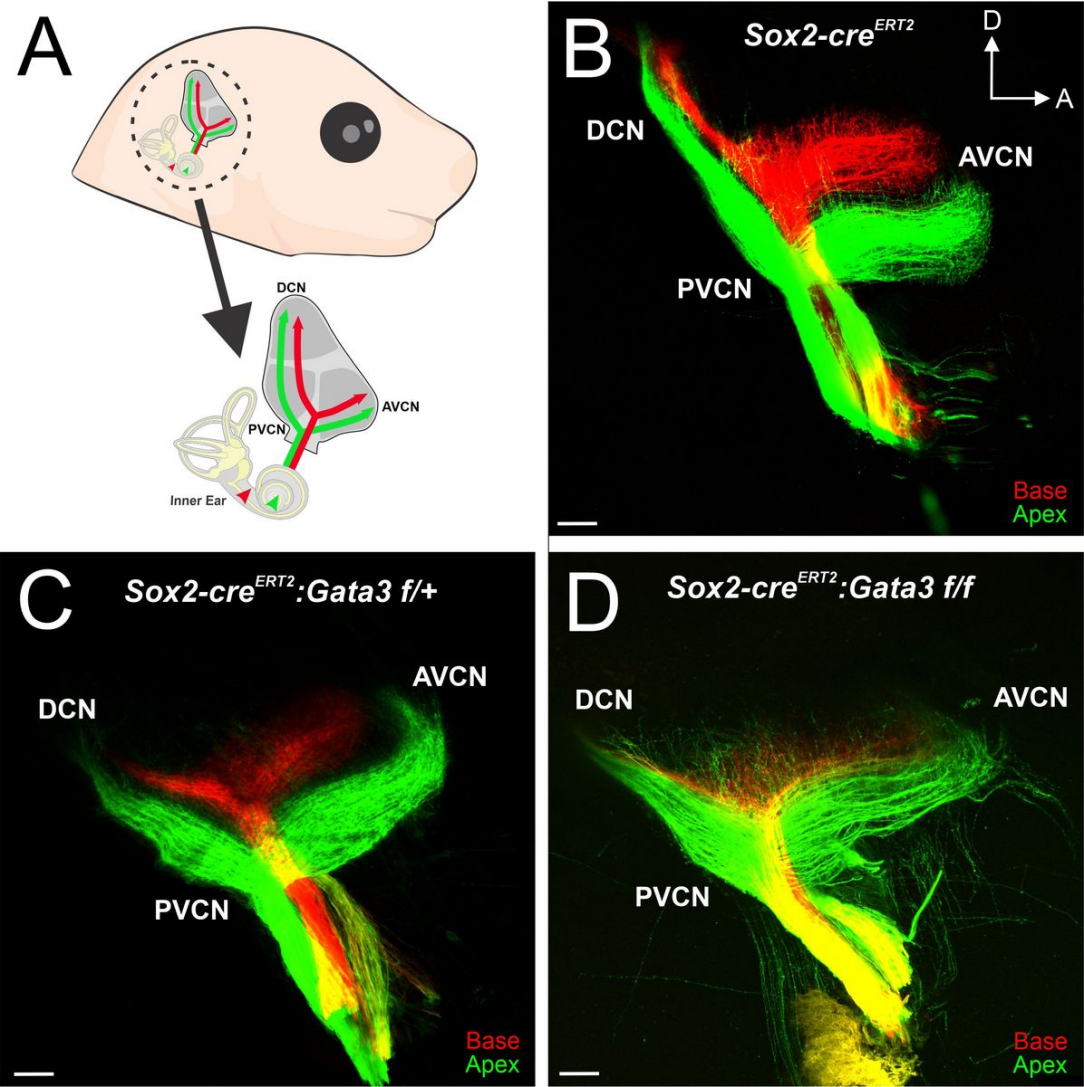
SGN central pathfinding unaffected by *Gata3* deletion (**A**) Schematic view of lipophilic dye placement and visualization of SGNs in the CN. (**B-D**) Lipophilic dye was applied to the base (red) and apex (green) of control and mutant cochlea at E18.5 and their central projections were analyzed. (**B**) In the *Sox2-cre*^*ERT2*^ control, SGNs bifurcate and send processes towards the AVCN and DCN/PVCN. Basal and apical SGN fibers also remain segregated throughout the CN (**C**) Heterozygous SGNs bifurcate and maintain tonotopic segregation similar to controls. (**D**) Homozygous mutants have aberrant SGN central projections with apical fibers projecting more dorsally and sometimes projecting outside the CN. Additionally, some SGN neurites project outside cranial nerve VIII before reaching the CN.

Given that homozygous mutants display aberrant peripheral projections of SGNs, with the phenotype progressively getting more severe in a basal to apical manner (Fig. 4), we next investigated whether central projection of SGNs to the cochlear nucleus (CN) was also affected. Previous studies examining the role of *Gata3* in spiral ganglion neuron central pathfinding have shown varied results depending on the location and timing of *Gata3* deletion^6,24^. Early deletion of *Gata3* throughout the entire inner ear at E9.5 results in central SGN fibers bifurcating at several branch points, with terminal fibers projecting non-specifically throughout the CN^6^. However, deletion of *Gata3* within delaminated SGNs at E9.5 results in normal projection of SGNs within the CN with tonotopy maintained^24^. Taken together these two studies suggest that *Gata3* may be affecting SGN neuron central pathfinding in a cell non-autonomous and time-dependent manner. In order to investigate this further, lipophilic dyes were applied to the base (red) and apex (green) of *Sox2-cre*^*ERT2*^ control, as well as heterozygous and homozygous mutant cochlea (Fig. 5A) to visualize the projections of SGNs into the CN. *Sox2-cre*^*ERT2*^ control SGNs entered the hindbrain and bifurcated sending ascending and descending process towards the anteroventral cochlear nucleus (AVCN) and dorsal cochlear nucleus (DCN)/posteroventral cochlear nucleus (PVCN) respectively (Fig. 5B). *Sox2-cre*^*ERT2*^ control SGNs remained segregated with basal fibers extending more dorsally and apical fibers more ventrally (Fig. 5B). This stereotyped central wiring was also maintained in heterozygous (Fig. 5C) mice. In contrast SGNs in homozygous mice display less segregation between apical and basal fibers. Apical fibers often project more dorsally into spaces occupied by basal fibers. Additionally, some apical fibers upon reaching the hindbrain project outside of cranial nerve VIII into areas outside of the CN (Fig. 5D). These results provide further evidence for the idea that *Gata3* expression plays an important role in the development and wiring of SGNs centrally. Our data along with previous studies^6,24^ suggest that Gata3 is acting in a cell non-autonomous manner at or before E11.5 to promote proper central wiring of SGNs. Further investigations are needed to elucidate what cell populations require early *Gata3* expression in order to promote proper central pathfinding of SGNs.

### Gata3 deletion at E11.5 results in full morphologic development of the cochlear duct and vestibular system, but shows progressive neurosensory epithelial loss and disorganization

**Figure 6:**
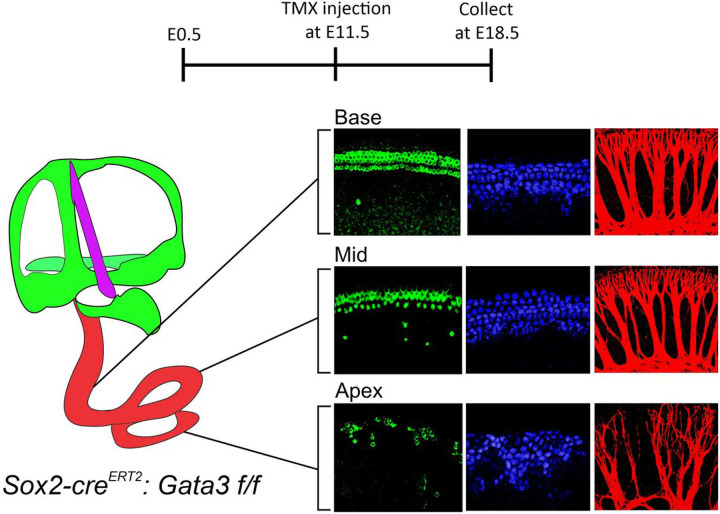
Loss of *Gata3* follows in the timeline of previous studies showing progressive basal to apical loss of sensory cells. Schematic of *Gata3*mutant ear at E18.5, indicating the cochlea (red), vestibular system (green), and endolymphatic duct (purple). Images are representative of the phenotype observed along the length of the cochlea and are taken from figures 2,3, and 4. Our study deleted *Gata3* later than previous studies at E11.5 and found that the overall morphology of the inner ear is intact unlike previous studies. In addition, there was a basal to apical loss of sensory cells indicating *Gata3* is still required for their formation and organization.

Previous *Gata3* deletion studies have shown a variety of phenotypes that include morphologic and cochlear neurosensory epithelia defects^5–7, 10,21,24,25^. *Gata3* null mice display a severely truncated cochlear and vestibular system which were devoid of sensory epithelia except for a small patch of HCs and SGNs in a portion of the saccule^5,7^. *Gata3* deletion at E8.5 using the *Foxg1-cre* mouse line resulted in a truncated cochlea which contained no HCs and abnormal morphologic development of the vestibular system^6^. *Gata3* deletion at E9.5 using the *Pax2-cre* mouse line resulted in similar morphologic defects including a truncated cochlea and abnormal vestibular system. However, deletion at E9.5 resulted in patchy sensory cell development of HCs, SCs, and SGNs^6,10^. In studies that have conditionally deleted *Gata3* from only SGNs, HCS and SCs form properly^24,25^. We contribute results for *Gata3* deletion at E11.5, a time in development in which proneurosensory cell differentiation is occurring. Our findings show that *Gata3* deletion at E11.5 results in a morphologically sound structure with a full length cochlea and well developed vestibular system (data not shown). Within the cochlea, the sensory cells in the OC are mostly present and have a varying phenotype depending on the cochlear region. In the homozygous mutant cochlear base, HCs and SCs are present with only mild disorganization (Fig. 6), while the homozygous mutant basal radial bundles have larger spacing than normal but the neurons are well organized. This contrasts the phenotype seen in the apex since the peripheral projection density of the mutant apex is decreased and those projections which are present appear disorganized (Fig. 6). Additionally, the tonotopy of SGN central projections is maintained within the CN in both heterozygous and homozygous mutants (Fig. 5). In comparison, the mutant apical HCs are severely reduced to patchy clusters with some ectopic HCs that appear in the GER, while the apical SCs are not organized in rows and instead cluster together (Fig. 6). Our data demonstrates a role for *Gata3* in all neurosensory cells after their initial specification.

## Discussion

*Gata3* was previously shown to be necessary for both proper morphology and cochlear neurosensory epithelia early in development when its expression is high throughout the entire otocyst^5–7, 10,21,39^. However, the role of *Gata3* in HC, SC, and SGN formation after its restriction to the proneurosensory region was unknown. Our study reveals novel findings that *Gata3* plays both a dose-dependent and necessary role in the formation and organization of neurosensory cell types, but does not have an impact on the overall morphology of the inner ear at this specific developmental time point.

This project contributes new knowledge about the role of *Gata3* on proneurosensory epithelia formation in a temporal window that fills a gap between previous studies that investigated *Gata3* deletion. From our results we find that deletion of *Gata3* from the proneurosensory domain at E11.5 results in a fully formed cochlear duct (data not shown). Regardless of the single or dual loss of *Gata3* alleles, both ears formed morphologically normal cochleas. Therefore, *Gata3* is not required for morphologic development at E11.5. Given that previous *Gata3* deletion studies did not see normal morphology of the cochlea^5,6^, it is intriguing that deletion of *Gata3* about two days later results in a morphologically sound inner ear with a fully formed cochlea. While HCs, SCs and SGNs do form, they are highly disorganized and this phenotype progressively worsens from base to apex. In *Gata3* heterozygous null mice, studies have found that OHC loss occurs without *Gata3*^12,21,40^. This phenotype is mirrored in our study, despite the difference in timing of which *Gata3* is deleted. It is also noteworthy that the heterozygous mutant had a subtler phenotype compared to the homozygous mutant, suggesting that precise levels of GATA3 are needed for proper formation and organization of the proneurosensory epithelia. If precise levels of *Gata3* are truly necessary, then increased levels of *Gata3* should also have a phenotype in our model. Several other over-expressor studies have been previously studied that demonstrated ectopic HCs in the GER^33–36, 38^. Previous studies have even used the *Gata3* over-expressor model in combination with upregulation of other sensory genes in order to increase the efficiency of ectopic HC formation^35,36^. Investigating the over-expression of *Gata3* in this model would be useful in determining the detrimental effects, if any, of higher levels of *Gata3* in the cochlea. While this would further elucidate the specific role of *Gata3* in the cochlea, the investigation of *Gata3* over-expression is especially pertinent since extra alleles of *Gata3* have also been known to cause HDR syndrome^20^.

Finally, it should also be noted that we deleted *Gata3* from three different cell types: HCs, SCs, and SGNs. While these cell types work together, it is unclear if loss of *Gata3* in just one of the cell types is enhancing the overall phenotype we see in our model. Despite the fact that HCs in our model are innervated by SGNs throughout the entire cochlea, we are unable to determine if the HC disorganization is causing the improper SGN peripheral projections when using the *Sox2-cre*^*ERT2*^ model, or vice versa. Likewise, since the SCs and HCs are connected via tight junctions in the normal OC, our model is unable to determine if a phenotype in one of these cell types is exacerbating the phenotype overall. Therefore, in order to tease apart the role of *Gata3* at this specific time point, future studies could use other more cell-specific Cre lines to delete *Gata3*. Comparison of our phenotype in this model to *Gata3* CKO in HC-specific, SC-specific, or SGN-specific lines could elucidate the exact role of *Gata3* in the proneurosensory stage of development.

In conclusion, our work demonstrates that *Gata3* is essential for proper cochlear neurosensory epithelia development and organization in the late proneurosensory stage at E11.5. Because our study demonstrates a phenotype in the heterozygous mutant in addition to a more severe phenotype in the homozygous mutant, we can confirm that correct levels of *Gata3* are also required for proper development. Furthermore, our study performs the latest embryonic *Gata3* deletion known in the field and contributes to the understanding that *Gata3* is required for proper formation and organization of the cochlea sensory epithelia at E11.5, but not for overall cochlea morphology.

## Methods

### Mouse model and genotyping

All animal care and procedures were approved by Western Michigan University Institutional Animal Care and Use Committee (IACUC) following the guidelines for use of laboratory animals (IACUC #20-11-01). All experiments were carried out in accordance with the ARRIVE guidelines, and all methods were carried out in compliance with all relevant regulations. The following mouse strains were used: *Sox2-cre*^*ERT2*^ (Jackson Labs)^26^, *tdTomato Ai9* (Jackson Labs)^41^, and *Gata3 Flox* were provided by Dr. Maxime Bouchard^42^. *Sox2-cre*^*ERT2*^ males were bred with *Gata3 f/f* females to produce males that were *Sox2-cre*^*ERT2*^: *Gata3 f/+*, who were viable. *Sox2-cre*^*ERT2*^: *Gata3 f/f* mice were produced by breeding *Sox2-cre*^*ERT2*^: *Gata3f/+* or *Sox2-cre*^*ERT2*^: *Gata3 f/f* males with *Gata3 f/f* or *Gata3 f/+* females. Genotyping was performed using the following primers: Cre 5’ CCT GTT TTG CAC GTT CAC CG 3’ and 5’ ATG CTT CTG TCC GTT TGC CG 3’ yield a 280 base pair (bp) mutant and IL2 5’ CTA GGC CAC AGA ATT GAA AGA TCT 3’ and 5’ GTA GGT GGA AAT TCT AGC ATC ATC C 3’ yield a 324 bp control band; *Gata3* 5’ GAT TCA GTC TCC CTC CTT CTT C 3’ yield a 430 bp mutant band and 5’ GTT CAC ACA CTC CCT GCC TTC TG 3’ yield a 400 bp control band; and *tdTomato Ai9* 5’ AAG GGA GCT GCA GTG GAG TA 3’and 5’ CCG AAA ATC TGT GGG AAG TC3’ yield a 297 bp WT band; 5’ CTG TTC CTG TAC GGC ATG G 3’ and 5’ GGC ATT AAA GCA GCG TAT CC 3’ yield a 196 bp mutant band. Breedings were performed with E0.5 specified as noon on the day of vaginal plug. Pregnant females received an intraperitoneal injection of 3 mg/40 g tamoxifen (TMX) and 2 mg/40 g progesterone at E11.5 between 9 and 11 am^30^. On the day of collection, the pregnant female was given a lethal intraperitoneal injection of Avertin (500 mg/kg 2.2.2-tribromoethanol). Embryos were dissected from the uterus, perfused with 4% paraformaldehyde (PFA) and stored at 4°C. All images are representative of at least three biological replicates.

### Whole-mount Immunohistochemistry

Whole mount immunohistochemistry was performed on previously fixed tissue^43^. Ears were washed in phosphate buffered saline (PBS), then washed five times five minutes in PBS/0.05% Tween20 followed by blocking for one hour in 5% normal donkey serum, 1% bovine serum albumin, and 0.5% TritonX-100 in PBS. The tissue was incubated in primary antibodies, diluted in blocking buffer, at 4°C for three nights. The following primary antibodies were used: MYO6 Rabbit (Sigma; 1:1000), MYOSIN7A Mouse (DSHB; 1:200), MYSOINVIIA Rabbit (Proteus Biosciences, Inc.; 1:500), Neurofilament 200 HC Chicken (Aves; 1:200), PROX1 Goat (R & D Systems; 1:200), and SOX2 Rabbit (Sigma; 1:500). Next, the tissue was washed four times thirty minutes, followed by overnight incubation at 4°C in secondary antibody in blocking buffer. Secondary antibodies were conjugated to Alexa flour anti-Mouse 488, anti-Rabbit 488, anti-Chicken 555, anti-Goat 647, or anti-Rabbit 647 (Life Tech; 1:1000). Nuclei were labeled using Hoescht Dye (1:2000), received as a gift from Bernd Fritzsch. Images were taken on either a Nikon C2 confocal microscope or a Leica Stellaris 5 confocal microscope and images were compiled in ImageJ and edited in CorelPhoto Paint (Version 19.0; 2017).

### Spiral Ganglion Neuron Quantification

For radial bundle quantification, shown in Fig. 4 and Supplementary Fig. S1 online, cochlea were imaged at the same magnification in the apex for each genotype. Using FIJI imaging software (Version 1.8.0_66), eight spaces between radial bundles were outlined. All area results were recorded in Graph Pad Prism (Version 9.1.2) and a TTEST analysis was performed. Data point plot graphs were constructed, and significance was set at P < 0.05.

### In situ hybridization

*Gata3* mRNA labeling was achieved using a previously described *in situ* hybridization protocol^43^. Mice were fixed in 4% PFA and inner ears were dissected in 0.4% PFA. Control ears and experimental ears were run together throughout the experiment to ensure both ears received the same experimental conditions. Ears were dehydrated overnight in 100% methanol and rehydrated through a graded methanol series. Ears were digested with Proteinase K in PBS (Ambion, Austin, TX, USA). Samples were hybridized overnight at 60°C to the *Gata3* riboprobe in hybridization solution consisting of 50% (v/v) formamide, 50% 2X saline sodium citrate (SSC), and 6% (w/v) dextran sulphate. Unbound probe was removed by performing washes with 2X SSC. Samples were then incubated with anti-digoxigenin antibody conjugated with alkaline phosphatase (Roche Diagnostics GmbH, Mannheim, Germany) overnight at room temperature. Ears were extensively washed with 1X washing buffer throughout the day, then left overnight in 1X washing buffer at room temperature. Samples were then incubated at room temperature in detection buffer (Roche) before being thoroughly saturated with nitroblue phosphate/5-bromo, 4-chloro, 3-indolil phosphate (BM purple substrate, Roche). Control and mutant samples were developed in BM purple for the same length of time. Ears were mounted in glycerol on a slide and imaged with a Nikon Eclipse E600 microscope and Canon EOS Rebel T7i camera. Images were edited in Corel Draw (version 19.0; 2017).

### Lipophilic Dye Tracing

Neuronal tracing of spiral ganglion neurons was conducted as previously described^43^. Briefly, the lateral half of the inner ear was exposed, and pieces of lipophilic dye-soaked paper was inserted into the base (NeuroVue^®^ Red) and apex (NeuroVue^®^ Maroon) of the cochlea. Heads were then placed into glass vials filled with 4% paraformaldehyde and incubated at 37°C for 3 days to allow for proper dye diffusion. Following incubation, the brains were removed, and the brain stem was flat mounted with the lateral side facing up in glycerol on a slide and imaged within 1 hour. All imaging was performed using a Leica Stellaris 5 confocal microscope with LAS X software and images were compiled in ImageJ and edited in CorelPhoto Paint (Version 19.0; 2017).

## Data Availability

Data is freely available upon request. Requests for data should be addressed to ZAS (zach.stoner@nih.gov).
